# Enhanced Biodegradation of Cyantraniliprole in Aqueous Systems by Novel Bacterial Consortia: Optimization, Degradation Efficiency, and Bioremediation Potential

**DOI:** 10.3390/microorganisms14061303

**Published:** 2026-06-09

**Authors:** Mohamed A. Fahmy, Shaza Y. A. Qattan, Rehab M. Baiomy, Belal M. Omar, Mohamed Maher, Mayasar I. Al-zaban, Khairiah M. Alwutayd, Osama K. Abou-Emera, Mohammed Aladhadh, Samir Mahgoub

**Affiliations:** 1 Department of Agricultural Microbiology, Faculty of Agriculture, Zagazig University, Zagazig 44511, Egypt; 2 Department of Biological Sciences, Faculty of Science, King Abdulaziz University, P.O. Box 80203, Jeddah 21589, Saudi Arabia; 3 Department of Biochemistry, Faculty of Agriculture, Zagazig University, Zagazig 44511, Egypt; 4 Department of Biology, College of Science, Princess Nourah bint Abdulrahman University, P.O. Box 84428, Riyadh 11671, Saudi Arabia; 5 Department of Animal and Poultry Production, College of Agriculture and Food, Qassim University, P.O. Box 6622, Buraydah 51452, Saudi Arabia; 6 Department of Poultry Breeding Research, Animal Production Research Institute, Agriculture Research Centre, Dokki, Giza 12618, Egypt; 7 Department of Food Science and Human Nutrition, College of Agriculture and Food, Qassim University, P.O. Box 6622, Buraydah 51452, Saudi Arabia

**Keywords:** cyantraniliprole, bacterial consortia, biodegradation, bioremediation, diamide pesticides, water treatment

## Abstract

This study aimed to isolate, characterize, and evaluate bacterial consortia capable of degrading the diamide insecticide cyantraniliprole in aqueous systems and to assess their bioremediation potential under environmentally relevant conditions. Four bacterial consortia, each comprising six isolates, demonstrated significant growth in mineral media containing cyantraniliprole as the sole carbon source, and the isolates were identified using conventional microbiological techniques in combination with MALDI-TOF-MS analysis. The bacterial consortia were enriched from pesticide-contaminated environments and systematically evaluated using microbiological, physiological, and analytical approaches to determine their degradation potential and environmental adaptability. The degradation performance of the consortia was systematically assessed under varying environmental parameters, including temperature, pH, salinity, and incubation time, with optimal degradation observed at 30–35 °C, pH 7.0–8.0, 0.5–5.0% NaCl, and 11 days of incubation at 150 rpm using an initial cyantraniliprole concentration of 50 mg/L. Biodegradation efficiency was further evaluated using DCPIP reduction assays, alongside measurements of biofilm formation and biomass production, indicating enhanced metabolic activity and adaptive responses under pesticide-induced stress. The consortia also exhibited the capacity to degrade structurally related diamide pesticides, including flubendiamide, chlorantraniliprole, cyclaniliprole, and fluchlordiniliprole, suggesting broad-spectrum biodegradation potential. Their performance was further validated in a simulated water microcosm system designed to mimic environmentally relevant contamination scenarios. In simulated contaminated water (60 mg/L cyantraniliprole), bacterial inoculants standardized to 10^7^ CFU/mL achieved substantial degradation after 20 days of incubation at 30 °C, as confirmed by HPLC analysis, with the six-strain consortium (T4), comprising *Bacillus subtilis* subsp. *subtilis* AZFS3, *Bacillus pumilus* AZFS5, *Bacillus mojavensis* AZFS15, *Bacillus paramycoides* AZFS18, *Pseudomonas aeruginosa* KZFS4, and *Alcaligenes aquatilis* KZFS11, demonstrating the highest removal efficiency (98.27%) and reducing the pesticide concentration to 1.00 mg/L, followed by consortium T3 (96.72%), which consisted of *Bacillus subtilis* Ht1, *Bacillus subtilis* Ht2, *Bacillus mojavensis* Ht3, *Pseudomonas aeruginosa* Ht4, *Pseudomonas aeruginosa* Ht5, and *Pseudomonas aeruginosa* Ht6. Residue analysis and predictive bioinformatic assessment further supported the biodegradation capacity of the selected bacterial communities and suggested the formation of simpler transformation products. Overall, the investigated bacterial consortia exhibited high degradation efficiency and environmental adaptability, highlighting their potential as effective and eco-friendly agents for the bioremediation of cyantraniliprole-contaminated water systems.

## 1. Introduction

Pesticides present a substantial threat to human health, with the potential to induce genomic mutations. These harmful compounds enter the human body through multiple pathways, including contaminated water, food chains, soil, air, and direct contact with plants, animals, and environmental health [1,2]. Of the pesticides applied, a mere 0.1% successfully target pests. The overwhelming majority, 99.9%, contaminate the environment by harming living organisms, disrupting enzyme systems, and reducing microbial diversity [3,4,5]. Insecticides rank among the most harmful of these chemicals, with global use totaling 4.19 million tons in 2019. This rising dependence on pesticides has led to extensive environmental pollution, creating significant risks for public health, natural resources, and economic stability. In response, bioremediation, a process that uses native microorganisms to break down pollutants, has become a promising, cost-effective, and eco-friendly strategy for mitigating pesticide contamination [6,7,8]. Diamide pesticides, such as flubendiamide (phthalic diamides) and cyantraniliprole, chlorantraniliprole, and tetraniliprole (anthranilic diamides), function with a specific mode of action. They selectively target the ryanodine receptors (RyR) in lepidopteran pests, such as moths and butterflies. This action induces uncontrolled muscle contractions, ultimately resulting in paralysis and death [9,10,11].

Cyantraniliprole (C_19_H_14_BrClN_6_O_2_), marketed by DuPont under the name Cyazypyr™, is a widely used insecticide. While research has extensively covered its efficacy, mode of action, and residue analysis, its environmental degradation pathways remain poorly understood. Consequently, there is a significant knowledge gap regarding its impact on microbial diversity and bacterial community composition [12,13]. The application of cyantraniliprole (CY), a widely used anthranilic diamide insecticide in viticulture, raises significant ecological concerns. While effective against sucking pests, this pest management method can result in the formation of metabolic byproducts that are often more toxic and persistent than the parent compound. A primary transformation product is the highly persistent metabolite IN-J9Z38, which presents a considerable environmental risk. Furthermore, studies on CY residues have demonstrated their adverse effects on key soil enzyme activities, notably suppressing dehydrogenase, alkaline phosphatase, and acid phosphatase. These inhibitory effects suggest that CY may have substantial consequences for soil health, particularly by disrupting the critical process of phosphorus mineralization [14,15]. The degradation pathway of Cyantraniliprole in soil was characterized, and several metabolites were identified, including IN-J9Z38, IN-JCZ38, IN-N7B69, and IN-QKV54. Among these, Cyantraniliprole was primarily transformed into IN-J9Z38, a highly persistent metabolite that may pose potential environmental concerns. In addition, bacterial isolates were recovered from Cyantraniliprole-enriched soil, and five isolates (CY3, CY4, CY9, CY11, and CY20) exhibited strong degradation capabilities, removing approximately 66–92% of Cyantraniliprole residues. These findings suggest that the identified bacterial strains have promising potential for bioremediation of pesticide-contaminated soils [14].

Bioremediation technologies are categorized according to whether the biodegradation of organic pollutants occurs in situ or ex situ, for instance, in bioreactors or compost systems [16]. Efficiency is enhanced through strategies like biostimulation, bioaugmentation, phytoremediation, and bioventing [17,18]. Microbial consortia are particularly effective due to their synergistic, multifunctional, and persistent nature, which allows for optimal substrate utilization [19]. Specifically, bioaugmentation introduces pollutant-degrading microbes into contaminated sites while also stimulating the indigenous microbiota [20]. Proven pesticide-degrading microbial agents include *Pseudomonas aeruginosa*, *Bacillus subtilis*, *Sphingomonas paucimobilis*, and several *Bacillus* species, all of which have demonstrated the ability to biodegrade various insecticides and other agricultural pesticides [21,22,23,24]. Xu, Xue [25], who demonstrated that the natural microbial consortium ACE-3 was capable of utilizing acetamiprid as the sole carbon and energy source. The authors further proposed a metabolic degradation pathway and investigated the consortium’s species diversity to elucidate the relationship between its structural composition and functional performance. Similarly, Zhang, Wang [26] reported that a microbial consortium achieved 90.49% degradation of bensulfuron under optimal conditions of pH 7, temperature of 20 °C, and an initial concentration of 20 mg/L. In addition, ref. [27] highlighted the remarkable efficiency of microbial consortia in the biodegradation of mixed pesticides, emphasizing their superiority over single-strain systems, as previously suggested by [28]. Moreover, mixed microbial consortia have demonstrated degradation efficiencies exceeding 90% for several pesticides, including atrazine, carbofuran, and glyphosate. For example, consortia consisting of *Ochrobactrum* spp. and Pseudomonas citronellolis exhibited enhanced biodegradation performance and metabolic versatility [29]. Likewise, ref. [30] reported that a bacterial consortium composed of *Proteus vulgaris*, *Vibrio* sp., *Serratia* sp., and *Acinetobacter* sp. showed significantly greater efficiency in degrading dichlorvos in fertilizer-amended soils compared with individual bacterial strains. The consortium was also capable of utilizing dichlorvos as the sole carbon source, demonstrating its strong potential for the bioremediation of pesticide-contaminated soils and aquatic environments. According to [24], microbial consortia demonstrate superior biodegradation efficiency due to the functional diversity and synergistic metabolic interactions among their constituent microorganisms. For example, a consortium composed of *Azospirillum*, *Cloacibacterium*, and *Ochrobacterium* completely degraded 50 mg L^−1^ glyphosate within 36 h. Recent advances in microbiome engineering have further broadened the potential of bioremediation through the targeted manipulation of microbial communities to enhance degradation specificity and efficiency. In one notable case, recombinant strains produced via protoplast fusion between *Psathyrella candolleana* and *Pseudomonas putida* achieved 78.98% degradation of pentachlorophenol in contaminated water. Collectively, these integrated strategies provide a sustainable and resilient approach for improving agroecosystem health and environmental remediation [24].

We hypothesized that bacterial consortia composed of indigenous pesticide-degrading strains could achieve enhanced biodegradation of cyantraniliprole through synergistic metabolic interactions and expanded enzymatic diversity. Specifically, this study aimed to: (i) isolate and identify efficient pesticide-degrading bacterial strains from contaminated Egyptian soils; (ii) construct and characterize three distinct six-member bacterial consortia (T1, T2, and T3) capable of degrading cyantraniliprole and other diamide pesticides, including chlorantraniliprole, flubendiamide, cyclaniliprole, and fluchlordiniliprole; (iii) optimize key abiotic factors influencing biodegradation efficiency, including temperature, pH, salinity, and incubation period; (iv) evaluate and compare the biodegradation performance of the three consortia under controlled laboratory conditions and in water microcosm systems artificially contaminated with 60 mg L^−1^ cyantraniliprole, in comparison with consortium T4, which previously demonstrated superior degradation of insecticides and herbicides in our earlier studies [23,31,32,33]; and (v) employ bioinformatic analyses to predict and elucidate the metabolic pathways involved in cyantraniliprole degradation. Ultimately, this study sought to identify highly efficient bacterial consortia capable of the sustainable biodegradation of cyantraniliprole and related diamide pesticides, thereby providing an eco-friendly strategy for mitigating pesticide pollution in aquatic environments.

## 2. Materials and Methods

### 2.1. Pesticides and Media

Cyantraniliprole (99.1% purity), along with Flubendiamide, Chlorantraniliprole, Cyclaniliprole, and Fluchlordiniliprole, was obtained from Sigma (Cairo, Egypt). A stock solution of each pesticide was prepared at a concentration of 1000 mg/L, following established methodologies [23,31,32]. The working concentrations added to the experimental media were adjusted according to the specific requirements of each assay. The mineral salt medium [34] was prepared with the following composition (in mg/L): K_2_HPO_4_ (500), KH_2_PO_4_ (250), NaCl (500), (NH_4_)_2_SO_4_ (230), CaCl_2_·2H_2_O (7.5), MgSO_4_·7H_2_O (100), MnSO_4_·7H_2_O (100), and FeCl_3_ (1). The final volume was brought to 1000 mL using distilled water, and the pH was adjusted to and maintained at 7.0, as described by Atlas and Synder [35]. Trypticase Soy Broth (TSB) was also procured from Sigma, Egypt.

### 2.2. Sampling, Enrichment, and Bacterial Isolation

Freshwater samples were collected from a site located near the Kafr El-Zayat Pesticides and Chemicals Factory (KZF), Egypt (30.8285° N, 31.8138° E), an area with a documented history of pesticide contamination. The adjacent section of the Nile River within the Kafr El-Zayat industrial zone is known to experience substantial pollution, particularly due to industrial activities and chemical discharges [36,37]. The samples were collected in 500 mL bottles and stored at 4 °C until analysis. For microbial enrichment, a Mineral Salt Medium was used, following the protocol by Atlas and Synder [35]. The physicochemical properties of the water samples, detailed in Table 1, were analyzed using standardized methods. These included two sample types: one for isolating Cyantraniliprole-degrading bacteria (Sample No. 1 from KZF) and another for subsequent bioremediation experiments (Sample No. 2), both from freshwater. The analytical methods were as follows: pH was measured with a Horizon Ecology Co. (Chicago, IL, USA) pH meter (Model 5995). Conductivity (EC) and total dissolved solids (TDS) were determined using a Y.S.I. Model 33 S-C-T Meter (Yellow Springs, OH, USA). Dissolved oxygen (DO) was quantified according to the method of Kopp and McKee (1983). Biological oxygen demand [38], reactive (ortho) phosphate, and calcium hardness (as CaCO_3_) were assessed via the EDTA titrimetric method, as described by the Standard Methods Association [39]. Total alkalinity and phenolphthalein alkalinity were measured following the method of Kumar and Ravindranath [40]. Chloride concentration was determined per the method of Ramteke and Moghe [41]. Nitrogen species were analyzed as follows: ammonia-nitrogen [42], Calcium, and Magnesium hardness [43], nitrate [44], and total nitrogen [45]. Phosphates (PO_4_) were measured according to EPA [46] guidelines. Magnesium hardness was analyzed using the method from Hawk, Oser [47]. Concentrations of heavy metals were determined as outlined by Ratnam, Jha [48].

For bacterial isolation, 10 mL aliquots of seawater samples were added to 100 mL of mineral salt medium [34] in 250 mL Erlenmeyer flasks, supplemented with 50 mg/L of Cyantraniliprole. Following the protocol of Setlhare, Kumar [49], the cultures were incubated at 30 °C with agitation at 150 rpm for one week. This enrichment process was repeated through successive sub-culturing into fresh MSM until stable microbial communities were established. Aliquots from these cultures were then spread onto MSM agar plates containing 50 mg/L Cyantraniliprole and incubated at 30 °C until colony growth was observed. Morphologically distinct colonies were purified by streaking onto Trypticase Soy Agar plates. Selected pure isolates were preserved as 20% (*v*/*v*) glycerol stocks at −70 °C for long-term storage. From the initial 45 isolates, 18 demonstrated the highest Cyantraniliprole degradation efficacy. The biodegradation capability of the 45 isolates was preliminarily assessed based on their ability to grow in mineral salt medium containing cyantraniliprole as the primary carbon source. Antagonistic interactions among the selected isolates were evaluated using the streaking method on nutrient agar plates. Isolates showing high biodegradation potential and no visible antagonistic activity were combined to construct the six-member bacterial consortia. These were used to construct three distinct consortia, each comprising six isolates. The members of each consortium were selected based on confirmed synergistic interactions, with assurance of no antagonism among them. The three most effective consortia were designated Consortium No. 1, No. 2, and No. 3. For comparative analysis, a previously characterized group, designated here as Consortium No. 4, was also included. This consortium, documented in our earlier studies for its proficiency in bioremediating diamide and other pesticides [23,31,32], served as a benchmark.

### 2.3. Characterization of Bacterial Isolates

Bacterial isolate identification was performed via Matrix-Assisted Laser Desorption Ionization-Time of Flight Mass Spectrometry (MALDI-TOF-MS) at the Academic Park research facility, Faculty of Medicine, Alexandria University, Egypt. Preparation of isolates involved suspending a biomass equivalent to half a 10-μL inoculating loop, comprising either large or aggregated smaller colonies, in 70% ethanol. The subsequent methodology adhered to established protocols for bacterial extraction, matrix preparation, target plate spotting, and system calibration. Spectra were generated using a Bruker MALDI-TOF MicroFlex LT mass spectrometer (Bremen, Germany) and analyzed with Bruker Biotyper software (version 2.0.4) [50,51,52,53].

Recent advances in mass spectrometry have significantly improved the speed, precision, and reliability of microbial identification, including both bacteria and fungi [54,55,56]. In the present study, MALDI-TOF-MS was employed to confirm the identity of selected pesticide-degrading bacterial isolates (Table 2 and Table 3). The Biotyper system identifies organisms by matching their protein mass spectra against a comprehensive database of reference strains, producing similarity scores; values below 1.700 require additional verification. This approach provides a rapid and highly specific method for bacterial identification compared with conventional techniques. Its efficiency and accuracy in microbial diagnostics have been widely demonstrated [53,57]. In addition, Bille, Dauphin [54] reported an identification accuracy of 99.2% (2609 out of 2630 isolates) for bacteria grown on solid media, confirming the robustness of this technique.

### 2.4. Bacterial Degradation of Cyantraniliprole Under Different Environmental Conditions

The degradation efficiency of the four bacterial consortia was evaluated across a range of environmental conditions, including temperature, pH, salinity, and incubation time, using established methodologies [58]. In each experiment, 1 mL of bacterial inoculum (10^7^ CFU/mL) was added to 250 mL of MS broth supplemented with 50 mg/L of cyantraniliprole, as previously described [58,59,60]. The inoculated flasks were incubated at 150 rpm under systematically varied parameters: temperatures of 25, 30, 35, and 40 °C; pH levels of 6.0, 7.0, 8.0, and 9.0; and NaCl concentrations of 0% (salt-free control), 0.5%, 2.5%, and 5.0%. Bacterial growth was monitored by measuring optical density at 600 nm (OD_600_) with a UV-2101/3101 PC spectrophotometer (Shimadzu, Kyoto, Japan) at 3, 7, 11, and 16-day intervals. A one-variable-at-a-time (OVAT) approach was employed for experimental optimization, wherein only the parameter of interest was altered, while others remained constant [49,61]. Uninoculated flasks served as negative controls throughout the study.

### 2.5. Assessment of Biofilm Formation by Cyantraniliprole-Degrading Bacteria

The four bacterial consortia’s capacity to form biofilms was evaluated using an adapted microtiter plate (MTP) assay, following established protocols [62,63]. Each consortium was cultured in a 96-well tissue culture plate with fresh Tryptic Soy Broth (TSB), supplemented with Cyantraniliprole as the sole carbon source at concentrations of 70, 80, 90, and 100 mg/L [64]. Control wells included a negative control (TSB without bacteria) and a positive control (TSB with bacteria, without insecticide). After a 24 h incubation at 35 °C to allow for biofilm formation, the initial biomass was assessed by measuring the optical density at 600 nm (OD_600_). The wells were subsequently rinsed gently with phosphate-buffered saline (PBS) to eliminate non-adherent cells, air-dried, fixed with methanol, and stained with crystal violet. Excess stain was removed by washing, and the bound dye was then solubilized using 95% ethanol. The biofilm biomass was quantified by measuring the absorbance of the solubilized crystal violet at 570 nm (OD_570_). A strain was deemed a biofilm producer if its absorbance exceeded the mean of the negative control by more than three standard deviations. Based on the criteria defined by Basson, Flemming [63], the strength of biofilm formation was categorized as follows: Weak: OD ≤ 2 × OD_NC. Moderate: 2 × OD_NC < OD ≤ 4 × OD_NC. Strong: OD > 4 × OD_NC. (where OD_NC represents the mean absorbance of the negative control). To maintain analytical precision, any absorbance values that deviated by more than four standard deviations from the mean were excluded from the analysis.

### 2.6. Determination of Cyantraniliprole (CPS) Biodegradation via 2,6-Dichlorophenol Indophenol (DCPIP) Decolorization

The biodegradation potential of bacterial strains for Cyantraniliprole (CPS) at 60 mg/L was assessed using a 2,6-dichlorophenol indophenol (DCPIP) assay. This method utilizes DCPIP as a redox indicator; its color transition from blue (oxidized) to colorless (reduced) signifies the acceptance of electrons from microbial metabolic processes [65]. Bacterial strains were initially cultured in Tryptic Soy Broth (TSB), harvested, washed, and resuspended in a fresh medium to standardize the cell density to an optical density (OD_600_) of 1.00. This standardized suspension served as the inoculum for test tubes containing Bushnell-Hass (BH) mineral medium, Cyantraniliprole, and 2,6-dichlorophenol indophenol (DCPIP), prepared according to established protocols [66,67]. The tubes were incubated at 35 °C with shaking. The time required for the complete decolorization of DCPIP was measured for each strain and consortium. The sample demonstrating the shortest decolorization time was identified as the most efficient degrader of Cyantraniliprole.

### 2.7. Biodegradation of Some Diamide Pesticides

The bacterial inoculum was prepared by culturing 50 mL of Tryptic Soy Broth (TSB) overnight at 30 °C on a rotary shaker at 150 rpm. After incubation, the cells were pelleted via centrifugation at 6000× *g* for 10 min and subsequently washed three times with 25 mL of sterile 0.0125 M phosphate buffer (pH 7.2) to ensure purity. The final inoculum was adjusted to 10^7^ CFU/mL. This standardized inoculum was then used to assess the capacity of four distinct bacterial consortia to degrade the diamide pesticides Cyantraniliprole, Chlorantraniliprole, Flubendiamide, Cyclaniliprole, and Fluchlordiniliprole, which served as the sole carbon and energy sources [23,32,58,64]. The experimental setup followed the methodologies [68,69] described. All experiments were performed in duplicate using 250 mL Erlenmeyer flasks containing mineral salt medium [34] amended with 100 mg/L of the target pesticide (cyantraniliprole, chlorantraniliprole, or flubendiamide), following established experimental protocols [23,32,70,71]. Each flask was inoculated with the standardized bacterial suspension (10^7^ CFU/mL) and incubated under controlled conditions to assess pesticide degradation efficiency. The culture conditions were maintained at pH 7.0 and 30 °C with a salinity of 0.5 g/L NaCl under continuous shaking at 150 rpm for an 11-day incubation period. Three control flasks containing no pesticide (either cyantraniliprole or other test compounds) were included in parallel to validate the experimental results. Following incubation, bacterial growth was quantitatively assessed by measuring optical density at 600 nm (OD_600_).

### 2.8. Bioremediation Analysis of Cyantraniliprole in Water

#### Water Microcosm System

The degradation of cyantraniliprole (CPS) in aqueous medium was analyzed using modified methodologies adapted from Wu, Li [72], Sims et al. (2019), and Fahmy, Salem [23] with some modifications. The bioremediation experiments assessed the degradation capacity of selected bacterial consortia in water-based systems. Four active bacterial consortia previously identified for diamide pesticide degradation were cultured in tryptic soy broth (TSB) at 30 °C for 24 h. These cultures were then combined to prepare composite inoculants, with the final bacterial concentration standardized to 10^7^ (CFU/dwt. mL) of water.

To prepare the inoculum, bacterial strains were cultured in 50 mL of tryptic soy broth (TSB) overnight at 30 °C under continuous shaking (150 rpm). After incubation, the cultures were centrifuged at 6000× *g* for 10 min to pellet the cells. The pellet was subsequently washed three times using 25 mL of sterile 0.0125 M phosphate buffer (pH 7.2) and resuspended to a standard concentration of 10^7^ CFU/mL. For the microcosm experiments, 20 mL of this bacterial suspension was introduced into each 500 mL water treatment. The experimental design consisted of eight different treatments, each replicated three times. All samples were incubated at 30 °C for 20 days to evaluate the kinetics of cyantraniliprole degradation.

In a simplified microcosm system, twelve liters of water were divided into 24 equal portions of 500 mL each. Three of these portions, designated as C0, served as negative controls and remained untreated, receiving no cyantraniliprole (CPS) contamination, microbial inoculum, or NPK nutrient supplementation. The remaining 21 portions were contaminated with the insecticide cyantraniliprole at a concentration of 60 mg/L.

The portions were placed in plastic jars with tightly sealed lids, each of which had a 15 cm diameter opening. Following the experimental design in Table 2, the jars were inoculated with bacteria according to the protocols established by Fahmy, Salem [23] and Fahmy, Attia [31].

Control treatments (C0, C1, and C2) received sterilized water instead of bacterial inoculum. The contents of each jar were mixed twice. The C: N:P ratio of the contents was adjusted to 100:15:1 using solutions of ammonium sulfate ((NH_4_)_2_SO_4_) and potassium dihydrogen phosphate (KH_2_PO_4_), as recommended by Sims, Sims [73] and following the methodology of Mukherjee, Das [74].

Each jar contained 500 mL of water, establishing a simplified microcosm system. To track environmental changes, periodic water quality analyses were performed throughout the study period using the following measurements:

### 2.9. Total Viable Bacteria Count of Cyantraniliprole-Degrading Bacteria

Total viable bacteria (TVB) were enumerated via the pour plate method according to established protocols of Toranzos, McFeters [75]. Sampling was performed at 0, 3, 7, 11, and 20-day intervals. For each treatment, a 1 mL aliquot was aseptically transferred to 9 mL of sterile 0.85% saline to create a serial dilution series (10^−1^ to 10^−6^). From each dilution, 0.1 mL was plated in duplicate on trypticase soy agar (TSA) and incubated at 30 °C for 48 h. Colonies on plates containing between 30 and 300 colonies were counted, and the bacterial population was expressed as colony-forming units per mL (CFU/mL), normalized to dry weight.

### 2.10. Cyantraniliprole (CPS) Residue Analysis

Water samples were randomly collected from all treatment groups at one hour post-application and again on days 3, 7, 11, and 20. Cyantraniliprole residues were extracted according to the QuEChERS method [76], which included a dispersive clean-up step. The resulting supernatant was carefully decanted into Eppendorf tubes for subsequent analysis via high-performance liquid chromatography (HPLC). Quantification of the residues was conducted at the Central Laboratory for Soil, Foods, and Feed (CLSFF), Faculty of Technology and Development, Zagazig University, using an ISCO Model 2350 HPLC absorbance detector. Chromatographic separation was achieved using a Kromasil^®^ 100-5-C18 column (15 cm length, 4.6 mm i.d., 8 mm o.d.) with an isocratic mobile phase of methanol and water (85:15, *v*/*v*) delivered at a flow rate of 1.2 mL min^−1^.

Calibration curves were generated using standard cyantraniliprole solutions, prepared via serial dilution of stock solutions to concentrations of 0.1, 0.5, 1.0, 5.0, 10.0, and 60.0 mg/L in methanol. The curve demonstrated exceptional linearity (R^2^ = 0.9999). Method accuracy was assessed through recovery studies, where fortified water samples (0.1, 0.5, and 1.0 mg/L) yielded recoveries of 96.47–96.60% across concentrations of 1, 2, 5, 10, and 60 mg L^−1^. No interfering peaks were detected under the optimized analytical conditions, confirming the method’s specificity and reliability.

### 2.11. Cyantraniliprole Pathway Prediction

Metabolic pathway inference was performed using a curated set of enzyme-catalyzed reactions from the EAWAG-BBD Pathway Prediction System. This data was augmented with structural and biochemical information from PubChem (https://pubchem.ncbi.nlm.nih.gov) and ChemSpider (http://www.chemspider.com) during Reactome analysis. To ensure consistency, all pathway predictions were generated from a standardized reference database. The University of Minnesota Pathway Prediction System (UM-PPS) utilizes an optimized, rule-based framework to predict diverse biotransformations. By integrating probability-weighted reaction likelihoods, the UM-PPS enhances prediction reliability for aerobic conditions, a methodology supported by prior research [23,31,58]. This synergistic approach, which combines mechanistic biochemical rules with empirical data, improves the accuracy of predicting metabolic pathways for environmental contaminants.

### 2.12. Statistical Analysis

Data were analyzed statistically with CoStat software, version 6.311. We performed a one-way analysis of variance (ANOVA) to compare treatment means, followed by Duncan’s multiple-range test for post hoc analysis, with a significance threshold of *p* < 0.05. Statistically homogeneous subsets are identified by common superscript letters in the results. To predict the degradation pathways of cyantraniliprole, we utilized ChemDraw 16, supplementing its analysis with molecular structures obtained from the PubChem and ChemSpider databases. For high-quality scientific visualization, all figures were generated using BioRender (https://www.biorender.com/) and GraphPad Prism, version 10.1.2.324.

## 3. Results

### 3.1. Identification of Cyantraniliprole-Degrading Bacteria

The bacterial isolates comprising each consortium were identified using a Bruker Daltonics MALDI-TOF-MS instrument, with the results detailed in Table 3. The composition of each consortium was as follows: Consortium No. 1 consisted of *B. subtilis* subsp. *subtilis* Re1, *B. cereus* Re2, *B. pumilus* Re3, *B. paramycoides* Re4, *B. mycoides* Re5, and *B. amyloliquefaciens* Re6. Consortium No. 2 was composed of *Serratia marcescens* Bo1, *Serratia marcescens* Bo2, *P. aeruginosa* Bo3, *P. aeruginosa* Bo4, *P. aeruginosa* Bo5, and *P. aeruginosa* Bo6. Consortium No. 3 contained *B. subtilis* Ht1, *Bacillus subtilis* Ht2, *B. mojavensis* Ht3, *P*. *aeruginosa* Ht4, *P. aeruginosa* Ht5, and *P. aeruginosa* Ht6.

### 3.2. Factors Influencing the Growth of Cyantraniliprole-Degrading Bacteria

#### 3.2.1. Effect of Temperature on Cyantraniliprole Biodegradation

**Figure 1:**
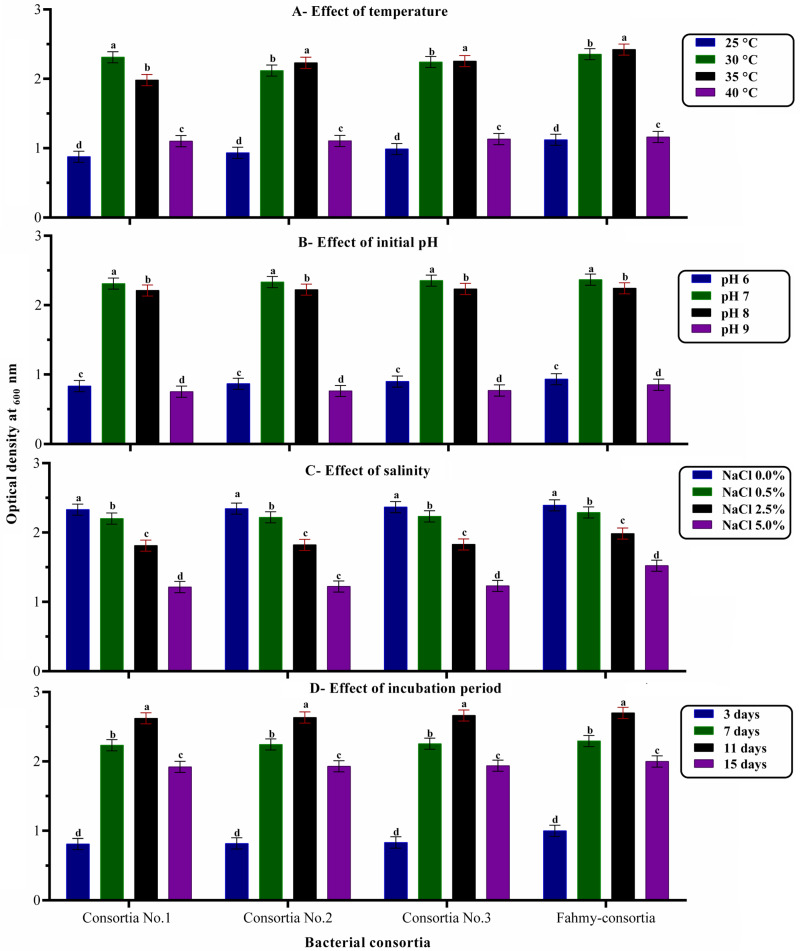
Bacterial growth of the tested 4 bacterial consortia on MS broth with cyantraniliprole at 50 mg/L ((**A**)—Effect of temperature, (**B**)—Effect of initial pH, (**C**)—Effect of salinity, and (**D**)—Effect of incubation period). Means and standard deviations of three replicates. Different letters on the bar indicate significant differences (*p* < 0.05).

Temperature is a key physicochemical factor that significantly influences the bioavailability and biodegradation of pesticides [59,77]. As depicted in Figure 1A, the growth of four bacterial consortia in Cyantraniliprole-amended MS broth was assessed across a gradient of incubation temperatures. Under controlled conditions (pH 7.0, 0.5% NaCl), growth, monitored by optical density at 600 nm over 11 days, was observed between 25 °C and 40 °C. Increasing the temperature from 25 °C to 40 °C resulted in a 2- to 3-fold rise in OD600, with optimal growth occurring at 30–35 °C. These findings indicate that moderately thermophilic conditions are most favorable for the biodegradation of cyantraniliprole by these bacterial consortia.

#### 3.2.2. Effect of Initial pH on Cyantraniliprole Biodegradation

As illustrated in Figure 1B, the initial pH of the medium is a critical factor governing both microbial growth and the degradation of Cyantraniliprole. The growth of the four bacterial consortia, measured via OD600 over an 11-day incubation at 30 °C and 0.5% NaCl, was profoundly affected by pH. Optimal growth occurred within a near-neutral to slightly alkaline range (pH 7.0–8.0), resulting in a two to three-fold increase in biomass. In contrast, growth was significantly inhibited at pH 9.0 and was minimal at pH 6.0. Among the consortia, Fahmy demonstrated the highest biomass production at pH 7.0, with consortium No. 3 being the next most prolific. These results suggest that a pH of 7.0–8.0 provides the optimal conditions for the microbial degradation of Cyantraniliprole, most likely by preserving vital enzymatic activity and supporting core metabolic functions.

#### 3.2.3. Effect of Salinity on Cyantraniliprole Biodegradation

To evaluate salinity tolerance, the bacterial consortia were cultivated in MS broth containing 0, 0.5, 2.5, and 5.0% NaCl under optimal conditions (30 °C, pH 7.0, and 50 mg/L Cyantraniliprole) for 11 days. As illustrated in Figure 1C, all consortia grew across the entire salinity range; however, biomass production declined at the highest concentration (5.0% NaCl). Optimal growth, corresponding to the highest biomass yield for all consortia, was observed at NaCl concentrations between 0.0% and 0.5%.

#### 3.2.4. Effect of Incubation Period on Cyantraniliprole Biodegradation

The effect of incubation time on bacterial growth was assessed over 3, 7, 11, and 15 days (Figure 1D) in MS broth (pH 7.0, 30 °C). Bacterial biomass increased steadily until day 11, demonstrating the consortia’s efficient use of Cyantraniliprole as a carbon and energy source. However, a pronounced decline in biomass was observed in all strains by day 15. This decrease was likely caused by nutrient depletion and the accumulation of toxic metabolic byproducts, which can inhibit further growth and degradation activity.

### 3.3. Effect of Bacterial Biofilm Formation on Cyantraniliprole Degradation

Bacterial consortia and environmental conditions influence biofilm morphology. Among the consortia tested, Fahmy-Consortia No. 4 demonstrated superior performance, achieving the highest biomass (OD_600_: 1.662–2.388) and biofilm formation (OD_570_: 0.593–0.792), exceeding the positive control values. Consortia No. 3, No. 2, and No. 1 also maintained robust biofilm production across all insecticide concentrations, with minimal signs of inhibition (Table 4).

### 3.4. Duration of DCPIP Indicator Decolorization

The degradation potential of individual bacterial consortia for cyantraniliprole (60 mg/L) was assessed by measuring the time to decolorization of the DCPIP redox indicator. The results revealed significant differences in degradation efficiency among the consortia (Table 5). Fahmy Consortium No. 4 demonstrated the most rapid degradation, with complete decolorization occurring within 17 h, indicating the highest degradation efficiency. Consortium No. 3 followed at 20 h, while Consortium No. 2 and Consortium No. 1 required 23 and 27 h, respectively. Overall, all four consortia exhibited rapid degradation, with decolorization times ranging from 17 to 27 h.

### 3.5. Biodegradation of Various Diamide Insecticides

The results of this experiment confirmed that all four tested bacterial consortia were capable of utilizing various diamide insecticides. To further evaluate their degradation potential, the study assessed their ability to break down five specific diamide pesticides, cyantraniliprole, flubendiamide, chlorantraniliprole, cyclaniliprole, and fluchlordiniliprole, proposed at a concentration of 100 mg/L as the sole carbon and energy source in a minimal salt medium [34] under optimized conditions.

Bacterial growth was monitored by measuring optical density at 600 nm (OD_600_) as an indirect indicator of bacterial adaptation and metabolic activity in pesticide-containing media. As detailed in Table 6, all consortia demonstrated robust growth across all tested insecticides, indicating their ability to survive and proliferate in the presence of these compounds. The recorded OD_600_ ranges were as follows: 2.144–2.211 for cyantraniliprole, 2.115–2.230 for flubendiamide, 2.166–2.272 for chlorantraniliprole, 2.198–2.262 for cyclaniliprole, and 2.112–2.216 for fluchlordiniliprole. Among the tested consortia, Fahmy Consortium No. 4 exhibited the highest growth, reaching an OD_600_ value of 2.272, followed by Consortium No. 3. These growth observations, together with the direct degradation results obtained in this study—particularly the high cyantraniliprole degradation efficiency achieved by Consortium No. 4—support its superior biodegradation potential and enhanced capacity for utilizing diamide insecticides under the tested conditions.

### 3.6. Water Microcosm System

#### 3.6.1. Bacterial Dynamics

**Figure 2:**
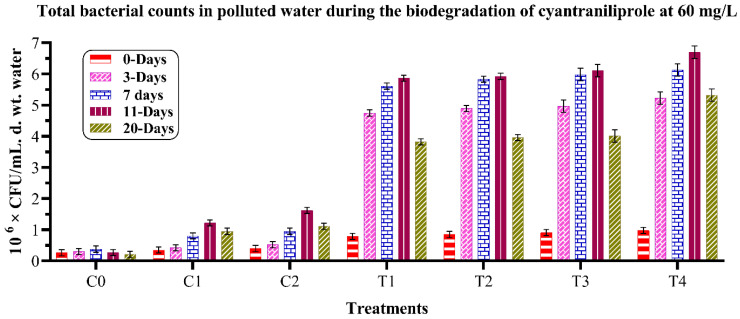
Total bacterial counts in water during the biodegradation of cyantraniliprole at 60 mg/L as affected by inoculation type with individual and/or mixed bacterial consortia during the incubation periods at 30 °C for 20 days. C0, water without any addition (No inoculum, No NPK, No CPS). Controls included C1 (cyantraniliprole-contaminated water without NPK or inoculum) and C2 (contaminated water amended with NPK but no inoculum). Experimental treatments (T1–T4) consisted of cyantraniliprole-polluted water supplemented with NPK and inoculated with distinct bacterial consortia: T1 (Consortium No. 1), T2 (Consortium No. 2), T3 (Consortium No. 3), and T4 (Consortium No. 4, Fahmy consortium).

As illustrated in Figure 2, the dynamics of viable bacterial populations in water were tracked over a 20-day cyantraniliprole biodegradation experiment. The untreated control group (C0) consistently demonstrated the lowest bacterial counts. Conversely, the experimental group supplemented with both nutrients and cyantraniliprole-polluted water (C2) promoted substantial growth of the native bacterial population. This group exhibited an increase from 0.40 to 1.62 × 10^6^ CFU/mL, a level of growth that surpassed that of the C1 treatment.

The bacterial consortia T1, T2, T3, and T4 all showed a consistent increase (*p* > 0.05) in total bacterial counts from the start of incubation to day 11, reaching 5.86 × 10^6^, 5.92 × 10^6^, 6.11 × 10^6^, and 6.70 × 10^6^ CFU/mL, respectively. After day 11, viable bacterial counts gradually declined across all treatments, reaching their lowest levels by day 20, coinciding with the complete depletion of cyantraniliprole. Among the treatments, T4 maintained the highest viable bacterial count, surpassing all controls (C0, C1, and C2), followed by T3, T2, and T1.

#### 3.6.2. HPLC Monitoring of Cyantraniliprole Biodegradation

**Figure 3:**
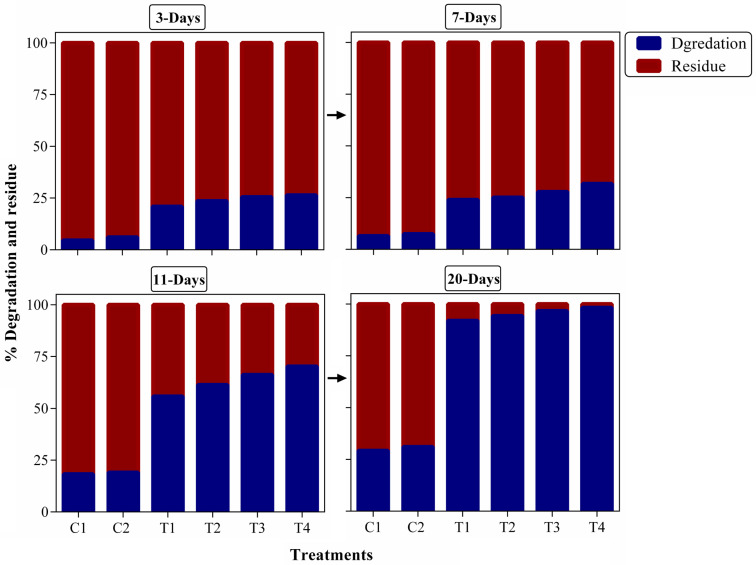
Biodegradation efficiency of cyantraniliprole (60 mg/kg in water) under the influence of different bacterial consortia over a 20-day incubation period at 30 °C, as quantified by HPLC. Controls included C1 (cyantraniliprole-contaminated water without NPK or inoculum) and C2 (contaminated water amended with NPK but no inoculum). Experimental treatments (T1–T4) consisted of cyantraniliprole-polluted water supplemented with NPK and inoculated with distinct bacterial consortia: T1 (Consortium No. 1), T2 (Consortium No. 2), T3 (Consortium No. 3), and T4 (Consortium No. 4, Fahmy consortium).

High-performance liquid chromatography (HPLC) was employed to quantify cyantraniliprole biodegradation by measuring its peak area in water samples. These samples were obtained from a bioremediation study in which water, initially contaminated with 60 mg/L of cyantraniliprole, was treated with individual or combined bacterial consortia. In the control groups (C1 and C2), degradation was limited, reaching only 29.27% and 31.11%, respectively, after a 20-day incubation period. Consequently, these groups exhibited high residual pesticide concentrations of 42.44 mg/L (70.73% remaining) and 41.33 mg/L (68.89% remaining), as illustrated in Figure 3.

In contrast, all bacterial consortia treatments (T1–T4) significantly enhanced biodegradation. After 20 days, the degradation rates reached 92.00%, 94.30%, 96.72%, and 98.27% for T1, T2, T3, and T4, respectively. These rates correspond to low residual concentrations of 4.64 mg/L, 3.30 mg/L, 1.90 mg/L, and 1.00 mg/L. The four-bacterial consortium (T4) demonstrated the highest degradation efficiency, likely due to synergistic interactions among its six active bacterial strains. It was closely followed in effectiveness by T3, T2, and T1. These findings indicate that these bacterial consortia represent a highly effective and environmentally sustainable solution for bioremediating water contaminated with cyantraniliprole, as presented in Table 7.

#### 3.6.3. The Metabolic Pathway of Cyantraniliprole: Key Reactions, Enzymes, and Predictive Modeling

**Figure 4:**
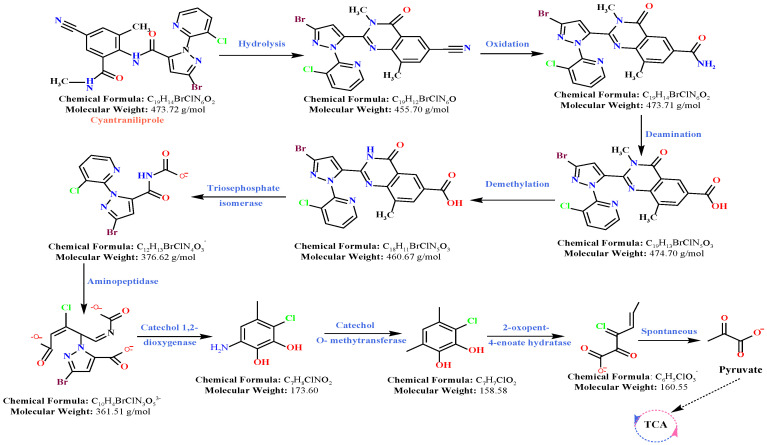
Modeling of Cyantraniliprole prediction pathway by the Swiss Federal Institute of Aquatic Science and Technology Biocatalysis/Biodegradation Database (EAW [AG-BBD) pathway prediction system.

Predicting the degradation pathway of cyantraniliprole is critically important, though such projections may not always align perfectly with conditions in natural environments. These predictive models serve as a valuable guide for researchers, directing future efforts to experimentally confirm and identify the insecticide’s degradation products. A bioinformatic analysis has proposed a 10-step degradation pathway. As illustrated in Figure 4, each step is catalyzed by a specific enzymatic reaction.

In step 1, Cyantraniliprole (C_19_H_14_BrClN_6_O_2_, MW 473.72) undergoes microbial degradation by a bacterial consortium. This process involves the reductive dehalogenation of the molecule’s cyanopyrazole ring, specifically removing the two hydrogen atoms and the oxygen atom from the amide group (-CONH_2_). This transformation results in the loss of H_2_O and a net removal of O_2_, forming the deoxy derivative, C_19_H_12_BrClN_6_O, with a reduced molecular weight of 455.70 g/mol.

In step 2, this conversion is an oxidation reaction catalyzed by bacterial enzymes. The process likely involves the addition of a hydroxyl group [78] to the molecule, followed by further oxidation. This introduces two hydrogen atoms and an oxygen atom (net addition of H_2_O), increasing the mass by 18 g/mol. The final step is the oxidation of an alcohol to a carboxylic acid, adding a second oxygen atom and resulting in the final formula and molecular weight of Cyantraniliprole (C_19_H_14_BrClN_6_O_2_, 473.71 g/mol).

In step 3, this transformation involves bacterial oxidative deamination. Enzymes likely target the cyano-amino group (-C≡N-NH_2_), replacing it with a carboxylic acid group (-COOH). This exchange substitutes a nitrogen atom for an oxygen atom and removes two hydrogen atoms. The net result is the loss of one nitrogen and one hydrogen and the gain of two oxygen atoms, leading to the new molecular formula and a slight increase in molecular weight.

In step 4, the bacterial consortia perform a demethylation reaction, specifically oxidative demethylation. This process targets a methyl ether group (-O-CH_3_) attached to the parent molecule. The bacteria enzymatically cleave the methyl group (CH_3_), converting the ether into a hydroxyl group [78]. This transformation results in the loss of a carbon and two hydrogen atoms (CH_2_), changing the formula from C_19_H_13_BrClN_5_O_3_ to C_18_H_11_BrClN_5_O_3_ and reducing the molecular weight by 14.03 g/mol.

In step 5, this biotransformation is a striking example of a retro-aldol cleavage, catalyzed by bacterial triosephosphate isomerase (TIM). While TIM’s primary role is in glycolysis, it can catalyze the reverse aldol reaction on xenobiotic compounds. The enzyme cleaves a carbon-carbon bond, breaking the large molecule into two fragments. The lost moiety (approximately C6H-2) is likely expelled, while the remaining structure undergoes tautomerization or rearrangement, potentially explaining the change in nitrogen count. This reaction is a critical detoxification or catabolic step, significantly reducing the molecule’s complexity and preparing it for further degradation by the microbial consortia.

In step 6, this conversion, facilitated by a bacterial aminopeptidase, involves the hydrolytic removal of an amino acid moiety. Aminopeptidases cleave peptide-like bonds at the N-terminus of a molecule. The transformation from C_12_H_13_BrClN_4_O_3_ to C_10_H_4_BrClN_3_O_5_ suggests the enzyme cleaved off a dipeptide or amino acid-derived fragment, such as glycine (C_2_H_5_NO_2_) or a similar unit. This hydrolysis reaction releases a small molecule and exposes a new terminal amine group on the core structure. The mass decrease of 15.11 g/mol and loss of a nitrogen atom are consistent with this enzymatic deamination, a common step in microbial degradation pathways.

In step 7, this describes the bacterial biodegradation of a brominated aromatic compound (C_10_H_4_BrClN_3_O_5_). A bacterial consortium first breaks down the complex molecule into a simpler intermediate catechol (C_6_H_6_O_2_). The enzyme catechol 1,2-dioxygenase then performs a key reaction: it cleaves catechol’s aromatic ring using molecular oxygen, inserting two oxygen atoms to form cis, cis-muconic acid. This critical step opens the ring structure, allowing the resulting linear molecule to be further degraded through central metabolic pathways into carbon dioxide and water, completing the detoxification process.

In step 8, while catechol O-methyltransferase (COMT) typically adds a methyl group, the mass change here shows a loss of 15 g/mol. This suggests a deamination reaction is the primary step. In bacterial consortia, multiple enzymes work sequentially. A deaminase likely first removes the amine group (NH_2_), forming an intermediate catechol. COMT then methylates this catechol, but this final methylation step is not reflected in the provided molecular weights, which only show the net loss from deamination.

In step 9, the bacterial enzyme 2-oxopent-4-enoate hydratase facilitates a key biodegradation step. It hydrates the chlorinated aromatic compound C_8_H_9_ClO_2_ (4-Chloro-2-methylphenoxyacetate degradative intermediate). This hydrolysis reaction adds a water molecule (H_2_O), breaking a double bond and incorporating oxygen and hydrogen. This transformation converts the linear C5 chain into a different structural isomer, ultimately yielding the smaller, rearranged molecule C_6_H_5_ClO_3_ (likely 2-Chloro-4-oxobut-2-enoate or similar), with a net loss of a C_2_H_4_ unit, explaining the change in molecular weight.

In step 10, the chlorinated aromatic compound C_6_H_5_ClO_3_- (molecular weight 160.55) is a key intermediate in microbial biodegradation. Bacterial consortia enzymatically cleave their ring structure, often via dioxygenase enzymes. This critical step transforms the molecule into a linear, aliphatic form. This linear intermediate is highly unstable and subsequently undergoes a spontaneous, non-enzymatic hydrolysis reaction. This rapid breakdown releases pyruvate, a fundamental metabolite that bacteria readily consume in the Krebs cycle for energy, completing the detoxification and assimilation of the original pollutant.

Pyruvate, a central metabolic intermediate, is actively transported into bacterial cells. Once inside, the pyruvate dehydrogenase complex catalyzes its oxidative decarboxylation. This reaction links glycolysis to the citric acid cycle (TCA) by producing acetyl-CoA. Acetyl-CoA is then carboxylated to oxaloacetate, priming the cycle. Within the TCA cycle, this substrate is systematically oxidized, generating reducing agents (NADH, FADH2), GTP, and CO_2_. This process provides the essential energy and precursor metabolites required for bacterial growth and function within the consortia.

**Figure 5:**
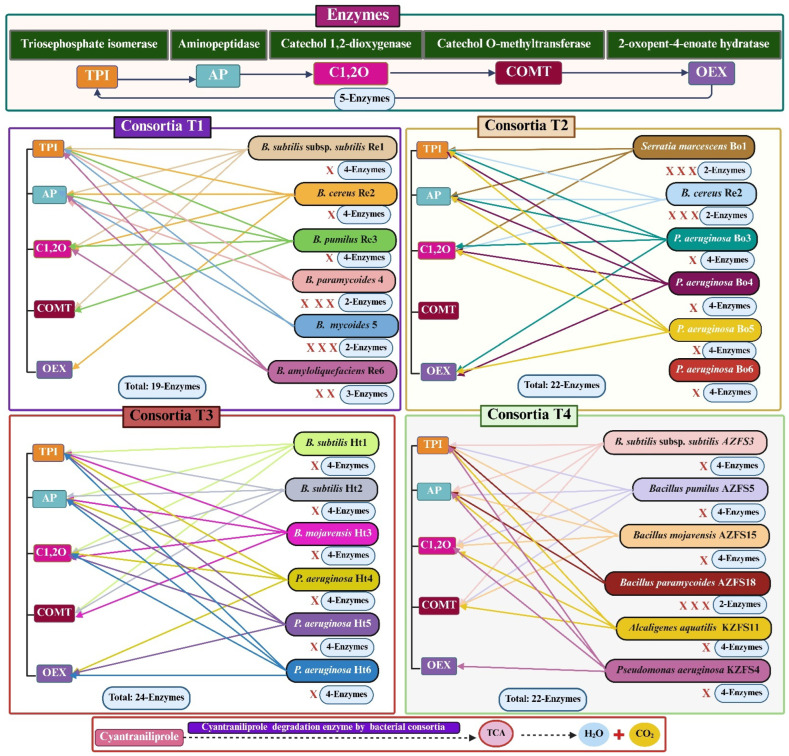
Bioinformatic analysis of bacterial contributions reveals enzyme activities of individual strains and consortia during cyantraniliprole biodegradation. **X** refers to inactivity. Consortia No. 1 contributed 19 enzymes, covering all 5. Consortia No. 2 contributed 22 enzymes, covering all 4 enzymes except Catechol O-methyltransferase (COMT). Consortia No. 3 contributed 24 enzymes, covering all 5. Consortia (T4) exhibited the most robust enzymatic activity, with 22 enzymes.

This study (Figure 5 and Table 8) of the four consortia evaluated for pesticide degradation efficiency showed that Consortia 3 (T3) and 4 (T4) demonstrated superior performance due to their comprehensive enzymatic profiles. Consortium 3 contributed a total of 24 enzymes, while Consortium 4 contributed 22; crucially, both possessed the complete set of five required enzymes, including catechol O-methyltransferase (COMT). In contrast, Consortium 2, despite having 22 enzymes, lacked COMT, and Consortium 1 contributed 19 enzymes but still covered all five. Bioinformatic analysis, performed after practical experiments, confirmed these findings and provided further insight. The data indicated that a modified tetra-strain consortium could potentially cover all necessary enzymes, suggesting that a simpler, four-member community might achieve efficiency comparable to the six-strain consortium. This presents a promising avenue for economizing the selection process in future applications. Subsequent studies should focus on experimentally validating this hypothesis.

## 4. Discussion

The widespread use of diamide pesticides raises significant environmental concerns due to their potential health and ecological risks. Consequently, the development of efficient bioremediation strategies is progressing to mitigate this form of pollution [10,79,80,81]. Cyantraniliprole, an anthranilic diamide insecticide widely employed in viticulture, represents a significant ecological threat. This is especially true for drench application methods, which result in the formation of metabolites that are both more toxic and more persistent than the parent compound [14,82].

In this study, maximum biomass of the bacterial consortium during cyantraniliprole degradation was observed at 30–35 °C, indicating a strong positive correlation between temperature and microbial activity. This finding aligns with previous research. Fahmy, Salem [32] also identified 30–35 °C as optimal for bacterial growth during diamide insecticide degradation, alongside other optimal conditions (pH 7.0–8.0, 0.0–0.5% NaCl, and 11 days of incubation at 150 rpm). Similarly, Lin, Chen [83] reported optimal cypermethrin degradation at 30–35 °C. According to Jadhav and David [64], optimal degradation of flubendiamide—achieving an 89.06% removal rate—occurred at 35 °C and a neutral pH of 7.0.

The thermal stability of a pesticide is principally governed by its molecular structure. Temperature modulates pesticide sorption by affecting two key properties: solubility and the rate of hydrolysis, processes influenced by solvation energy (G) and hydrolysis rate constants [84]. Optimal microbial proliferation and metabolic activity, essential for biodegradation, occur within a physiological temperature range of 25–35 °C. Accordingly, pesticide degradation is most efficient under mesophilic conditions, typically between 25 °C and 40 °C [85]. This is supported by studies indicating that 15–40 °C is a suitable range for biodegradation by specialized bacterial strains [84,86]. Qingyan, Ying [85] reported a 95.0% removal rate for atrazine at 30 °C from an initial concentration of 500 mg L^−1^. This aligns with findings for other pesticides, including carbofuran, chlorpyrifos, and DDT, which also exhibit peak degradation efficiency within the 25–30 °C range [86,87,88,89]. This specific thermal range is highly conducive to the metabolism of prevalent pesticide-degrading bacteria, such as species within the *Pseudomonas*, *Bacillus*, and *Alcaligenes* genera [87,88,90].

The optimal pH for both bacterial growth and degradation was observed to be between 7.0 and 8.0, indicating a strong pH dependence. This finding aligns with previous reports on endosulfan degradation, rhamnolipid production, and biosurfactant activity [91,92,93,94,95]. In contrast, deviations from this optimum to acidic or alkaline extremes (pH 6.0 or 9.0) reduced biomass, an effect attributed to impaired metabolic and enzymatic function, as supported by prior literature [32,96,97].

Maximum growth of the bacterial consortia was observed at low NaCl concentrations (0.0–0.5%). A clear correlation was observed between salinity, microbial activity, and pesticide degradation efficiency. This aligns with the established role of salts in facilitating diamide pesticide hydrolysis and with reports that salinity modulates pesticide solubility and microbial enzyme function [23,32,98]. The observed decline in degradation efficiency under saline conditions aligns with Yun, Ro [99], who reported that salinity impairs pesticide solubility and inhibits enzyme activity.

The bacterial biomass peaked on day 11, indicating highly efficient use of the substrate. However, a subsequent decline by day 15 suggests this growth was likely limited by nutrient exhaustion or the inhibitory effects of toxic metabolic by-products. This growth-and-degradation trajectory is consistent with patterns documented in similar research. Comparative studies have reported analogous degradation efficiencies: Hussain, Arshad [100] achieved 22–93% endosulfan removal over 14 days, and Sharma, Saxena [101] noted 71.6% chlorpyrifos (CPS) degradation by a *Bacillus*/*Micrococcus* consortium within 10 days. Furthermore, *Staphylococcus* aureus has been shown to degrade 80% of CPS in 14 days. In a study on a different pesticide, Doolotkeldieva, Konurbaeva [102] also reported potent aldrin degradation by both individual and consortium cultures of *Bacillus polymyxa* and *Pseudomonas fluorescens* within 12 days.

The four bacterial consortia tested, Consortium No. 4 yielded the highest biomass, followed by Consortium No. 3. All four demonstrated the capacity to use cyantraniliprole as their sole carbon source for growth and, importantly, to form biofilms—a critical trait for bioremediation applications. Biofilm morphology varies based on bacterial composition and environmental conditions. This ability to form robust, stress-resistant biofilms significantly increases their potential for practical use in decontaminating polluted sites. These findings align with the established model of biofilm development, which proceeds through initial attachment, microcolony formation, and maturation into complex three-dimensional structures reinforced by extracellular polymeric substances (EPS) [103,104]. Whereas traditional pesticide degradation research has primarily focused on free-floating (planktonic) bacteria, this approach fails to capture the major advantages of biofilms, which provide greater resistance to toxins and enhanced metabolic efficiency [105]. The fact that these consortia formed biofilms directly on cyantraniliprole, even at a high concentration of 100 mg/L, underscores their adaptability and proficiency in degrading the pesticide. This supports previous work by Cycoń, Wójcik [96] and Lima, Moreira [106] on microbial survival strategies, confirming that biofilm formation is a key mechanism governing degradation kinetics. Overall, the results highlight the superior bioremediation potential of bacterial consortia in contaminated environments, advancing beyond the constraints of conventional liquid culture studies. The bioremediation capabilities of *B. subtilis*, for instance, are not limited to liquid cultures; its biofilms generate protective matrices [107] and maintain growth even under pesticide-induced stress [108,109,110].

The time required for DCPIP decolorization, which indicates the rate of cyantraniliprole degradation, varied among the bacterial consortia, ranging from 17 to 27 h (Table 5). The Fahmy consortium demonstrated the fastest degradation activity, decolorizing DCPIP in 17 h, closely followed by Consortium No. 3 at 20 h. In contrast, Consortium No. 1 and No. 2 required 27 and 23 h, respectively. This variation in decolorization times aligns with the findings of Bidoia, Montagnolli [111], who established a correlation between rapid DCPIP reduction and high hydrocarbon degradation efficiency. The assay confirms that DCPIP serves as an efficient and low-cost indicator of microbial biodegradation activity [66,67,112], where the decolorization rate is directly proportional to the microorganisms’ metabolic prowess. The demonstrated capability of the tested hydrocarbon-degrading strains, including *P. aeruginosa*, *B. subtilis*, *S. odorifera*, *Micrococcus* spp., and *F. aquatile* [113,114], rapidly reduces DCPIP, further confirming their significant potential for bioremediation applications.

All tested microbial consortia demonstrated the ability to effectively degrade a mixture of diamide pesticides, cyantraniliprole, flubendiamide, chlorantraniliprole, cyclaniliprole, and Fluchlordiniliprole, each at a concentration of 100 mg/L in a minimal salt medium [34]. Among them, Fahmy-Consortia exhibited superior performance, achieving an optical density (OD600) of 2.211–2.272, followed closely by Consortia No. 3 (OD600 2.188–2.262). These high OD readings indicate robust microbial growth and enhanced metabolic activity. This finding, that consortia are more effective than single bacterial strains at degrading pesticides, is consistent with established research. For instance, it corroborates the work of Kadhim, Rabee [115], who reported a 98.32% degradation rate for malathion using a consortium. Microbial consortia are particularly advantageous in natural environments due to their collective metabolic versatility, enabling them to break down multiple pesticide types [116,117,118,119]. This contrasts with many study approaches that focus on isolating individual microorganisms to assess their degradation capabilities in isolation [58,64,120,121].

Microcosm experiments revealed a decline in bacterial consortia viability after 20 days, likely attributable to toxin accumulation or nutrient depletion [122]. Interestingly, positive controls (C1/C2) sustained higher viability than the untreated control (C0), suggesting that cyantraniliprole may stimulate microbial adaptation. Nutrient supplementation was identified as a critical factor in enhancing cyantraniliprole biodegradation. Maintaining a balanced C: N: P ratio, achieved by adding ammonia and phosphate, significantly accelerated pesticide breakdown, as demonstrated by Huang, Xiao [123]. Among the treatments, consortium T4 exhibited the highest viability, followed by T3, both outperforming T1 and T2.

The consortia tested, T1 through T4, proved to be highly effective for bioaugmentation and the removal of diamide pesticides from water. This superior performance is attributed to the synergistic relationships among the six constituent bacterial species, which together increased both the speed and the completeness of cyantraniliprole biodegradation. These results are consistent with the work of Zhang, Wu [124], who noted that successful bioremediation in natural settings usually relies on microbial consortia rather than single species, as different strains fulfill specialized functional roles. Previous studies further corroborate the advantage of a consortium over a single bacterium [125,126,127], which observed that individual strains rarely achieve complete degradation of pollutants. By integrating bacteria with complementary metabolic capabilities, a consortium harnesses the unique strengths of each member, resulting in more efficient and resilient pollutant breakdown. Moreover, mixed microbial consortia demonstrate greater substrate tolerance and enhance overall degradation, offering distinct advantages over the application of single strains.

Using the QuEChERS extraction method and HPLC [76], quantified cyantraniliprole residues. The control treatments (C1 and C2) exhibited minimal degradation. However, the marginally higher activity in C2 suggests that nutrient addition enhanced the native microbial community’s metabolic rate. The limited degradation in both controls is likely attributable to the inherent biodegradability of cyantraniliprole by indigenous water microorganisms; this efficiency was further enhanced by nutrients in the C2 treatment. All identified intermediates matched authentic cyantraniliprole standards.

The high degradation efficiency of the consortia underscores their potential for remediating persistent pesticides like cyantraniliprole, which poses significant ecological risks [14]. Consortium T4 achieved 98.27% degradation of the initial 60 mg/kg concentration within 20 days, followed by T3 (96.72%), T2 (94.30%), and T1 (92.00%).

This finding aligns with prior studies on microbial consortia for pesticide biodegradation. Xu, Xue [25] gradually acclimated a natural microbial consortium (ACE-3) that used acetamiprid as its sole carbon and energy source. By identifying intermediate compounds, the authors suggested potential metabolic pathways for acetamiprid degradation and examined shifts in community structure. Further supporting the effectiveness of consortia, Zhang, Wang [26] described a bacterial consortium that degraded 90.49% of bensulfuron methyl within 20 days under optimal conditions. Similarly, Levío-Raimán, Bornhardt [27] showed that a formulated bacterial consortium improved the degradation of a mixture of iprodione and chlorpyrifos. Formulated consortia often demonstrate greater productivity and resilience than single strains, making them a promising bioremediation strategy [28]. For instance, consortia have been reported to degrade pesticides such as atrazine, carbofuran, and glyphosate with efficiencies exceeding 90%. Notably, Góngora-Echeverría, García-Escalante [29] observed the highest degradation rates using a consortium of *Ochrobactrum* and *Pseudomonas* strains. In a study focusing on organophosphate pesticides, Agarry, Olu-Arotiowa [30] found that both a bacterial consortium and four isolated strains could utilize dichlorvos as a sole carbon source. The consortium achieved the highest dichlorvos removal efficiency when supplemented with NPK fertilizer, outperforming treatments with other nutrient sources. This indicates considerable potential for bioremediating soil and water contaminated with organophosphates. The consortium, characterized through morphological and biochemical methods, was tentatively composed of strains identified as *Proteus vulgaris*, *Vibrio* sp., *Serratia* sp., and *Acinetobacter* sp.

Advancements in bioinformatics are strengthening the design and application of microbial consortia by allowing scientists to predict the metabolic pathways involved in degrading cyantraniliprole. Research on related compounds like chlorantraniliprole [128] has shown that amidase-mediated pathways are critical for this degradation [23,58]. This aligns with the work of Das, Shafi [129], who highlighted the importance of using computational tools to predict xenobiotic metabolism and optimize bioremediation strategies.

The four microbial consortia tested for their ability to degrade the diamide pesticides T3 and T4 performed best. This superior efficacy is directly linked to their robust enzymatic makeup. While T3 had 24 enzymes and T4 had 22, the critical factor was that both contained all five key enzymes necessary for the complete degradation pathway, most notably including the enzyme catechol O-methyltransferase (COMT). The other consortia highlight the importance of this full suite. T2, which also had 22 total enzymes, lacked COMT, and this deficiency likely impaired its performance. Conversely, T1, despite having the lowest total (19 enzymes), still possessed all five essential enzymes, though its lower overall count may have limited its efficiency. Bioinformatics performed after the lab experiments confirmed these findings. They provided a significant new insight: the data imply that a carefully selected consortium of just four bacterial strains could, in theory, produce all the required enzymes. This suggests a simpler, four-member community could be as effective as the six-strain one used in this study. This revelation is promising for future applications, as it could significantly simplify and reduce the cost of developing effective bioremediation solutions. However, this hypothesis must be confirmed through practical experimental validation in subsequent research.

To facilitate practical bioremediation, subsequent studies should prioritize the characterization of bacterial enzymes that degrade cyantraniliprole. It is equally important to elucidate how environmental factors, such as nutrient availability, oxygen concentration, and the physicochemical properties of pesticides, influence biodegradation in natural settings. Furthermore, thorough economic assessments are indispensable for developing scalable and cost-effective strategies suited for real-world implementation.

## 5. Conclusions

Based on the study findings, the developed bacterial consortia, particularly T3 and T4, demonstrated strong potential for the bioremediation of cyantraniliprole and other diamide insecticides. The results indicated that optimal degradation occurred at mesophilic temperatures (30–35 °C), a neutral to slightly alkaline pH (7.0–8.0), low salinity, and an incubation period of approximately 11 days. These environmental conditions significantly enhanced microbial growth, biomass production, and overall degradation efficiency, highlighting their critical role in regulating pesticide biodegradation. All tested consortia exhibited strong biofilm-forming ability, which increased with higher pesticide concentrations. This enhanced biofilm production contributed to improved survival, stress tolerance, and degradation performance. Among the tested groups, T4 showed the highest biomass accumulation, strongest biofilm formation, and the fastest metabolic activity, as indicated by rapid DCPIP decolorization (17 h), followed closely by T3 (20 h). Furthermore, all consortia were capable of utilizing multiple diamide insecticides as sole carbon sources, confirming their broad metabolic adaptability. In microcosm experiments, cyantraniliprole degradation efficiencies ranged from 92.00% to 98.27%, with T4 achieving the lowest residual concentration (1.00 mg/L) after 20 days. Bioinformatic analysis further suggested a multi-step enzymatic pathway responsible for efficient pesticide transformation and detoxification. Overall, the findings clearly demonstrate that microbial consortia provide a highly effective and eco-friendly strategy for degrading persistent diamide insecticides, outperforming single-strain approaches through synergistic interactions and functional complementarity.

## Figures and Tables

**Table 1:** Physicochemical analysis of water used for isolation water, Sample No. 1, and for microcosm experiment water, Sample No. 2.

Physiochemical Analysis	Sample No. 1	Sample No. 2	Unites
(pH)	7.6 ± 0.2	7.57 ± 0.2	
Sodium (Na)	25.66	20.90	mg/L
Potassium (K)	13.93	11.44	
Cadmium (Cd)	0.45 ± 0.02	ND	
Iron (Fe)	0.11 ± 0.02	0.01 ± 0.02	
Magnesium	15.9	6.77	
Lead (Pb)	0.34 ± 0.02	ND	
Zinc (Zn)	0.22 ± 0.05	0.12 ± 0.14	
Manganese (Mn)	0.03 ± 0.01	0.02 ± 0.01	
Dissolved oxygen (DO)	6.87 ± 0.2	6.11 ± 0.2	
Biochemical oxygen demands	2.9 ± 0.02	2.3 ± 0.02	
Total alkalinity (T. ALK)	250 ± 0.2	150 ± 0.2	
Conductivity (EC)	344 ± 0.2	302 ± 0.2	µS/cm
Total dissolved salts (T.D.S)	0.892 ± 0.06	0.792 ± 0.06	g/L
Chlorides	17.54 ± 0.25	15.43 ± 0.23	
Calcium hardness	1.02 ± 0.03	1.00 ± 0.03	
Magnesium hardness	1.0 ± 0.02	0.2 ± 0.02	
HCO_3_	ND	ND	mg/L
Phosphates (PO_4_)	0.1 ± 0.02	0.03 ± 0.02	
Nitrate	0.21 ± 0.01	0.016 ± 0.01	

**Table 2:** The experimental design of the bioremediation treatment was used in microcosm systems.

Treatment	NPK	Inoculum Type	CPS	Inoculum Content
C0	−	Native bacteria	−	Negative control
C1	−	Native bacteria	+	Positive control-1
C2	+	Native bacteria	+	Positive control-2
T1	+	Consortia No. 1	+	*B. subtilis* subsp. *subtilis* Re1, *B. cereus* Re2, *Bacillus pumilus* Re3, *Bacillus paramycoides* Re4, *Bacillus mycoides* Re5, and *B. amyloliquefaciens* Re6
T2	+	Consortia No. 2	+	*Serratia marcescens* Bo1, *Serratia marcescens* Bo2, *Pseudomonas aeruginosa* Bo3, *Pseudomonas aeruginosa* Bo4, *Pseudomonas aeruginosa* Bo5, and *Pseudomonas aeruginosa* Bo6
T3	+	Consortia No. 3	+	*Bacillus subtilis* Ht1, *Bacillus subtilis* Ht2, *Bacillus mojavensis* Ht3, *Pseudomonas aeruginosa* Ht4, *Pseudomonas aeruginosa* Ht5, and *Pseudomonas aeruginosa* Ht6
T4	+	Fahmy-consortia	+	*Bacillus subtilis* subsp. *subtilis* AZFS3, *Bacillus pumilus* AZFS5, *Bacillus mojavensis* AZFS15, *Bacillus paramycoides* AZFS18, *Pseudomonas aeruginosa* KZFS4, and *Alcaligenes aquatilis* KZFS11 [32]

**Table 3:** Bruker Daltonik MALDI Biotyper rates classification results.

No.	Isolate Code	Analyte Name	Organism (Best Match)	Score Value
1	Consortia No. 1	Re1 (+++) (A)	*B. subtilis* subsp. *subtilis* DSM 10	2.335
		Re2 (+++) (A)	*B. cereus* DSM 31	2.333
		Re3 (+++) (A)	*Bacillus pumilus* ATCC 7061	2.344
		Re4 (+++) (A)	*Bacillus paramycoides* HS-1	2.321
		Re5 (+++) (A)	*Bacillus mycoides* DSM 2048	2.359
		Re6 (+++) (A)	*B. amyloliquefaciens* DSM 7	2.381
2	Consortia No. 2	Bo1 (+++) (A)	*Serratia marcescens* ZCF25	2.393
		Bo2 (+++) (A)	*Serratia marcescens* UCP 1549	2.377
		Bo3 (+++) (A)	*Pseudomonas aeruginosa* MTCC 4996	2.375
		Bo4 (+++) (A)	*Pseudomonas aeruginosa* 47T2	2.322
		Bo5 (+++) (A)	*Pseudomonas aeruginosa* IITG21	2.323
		Bo6 (+++) (A)	*Pseudomonas aeruginosa* dsvp20	2.326
3	Consortia No. 3	Ht1 (+++) (A)	*Bacillus subtilis* MTCC 441	2.325
		Ht2 (+++) (A)	*Bacillus subtilis* IH-1	2.329
		Ht3 (+++) (A)	*Bacillus mojavensis* B1811	2.330
		Ht4 (+++) (A)	*pseudomonas aeruginosa* cctcc ab93066	2.341
		Ht5 (+++) (A)	*Pseudomonas aeruginosa* PA1	2.363
		Ht6 (+++) (A)	*Pseudomonas aeruginosa* NCIM 5514	2.349
Comparison of consortia identified from Fahmy, Salem [32]				
4	Fahmy-Consortia	AZFS3	*Bacillus subtilis* subsp. *subtilis* AZFS3	LC599401.1
		AZFS5	*Bacillus pumilus* AZFS5	LC599402.1
		AZFS15	*Bacillus mojavensis* AZFS15	LC599403.1
		AZFS18	*Bacillus paramycoides* AZFS18	LC599406.1
		KZFS4	*Pseudomonas aeruginosa* KZFS4	LC599404.1
		KZFS11	*Alcaligenes aquatilis* KZFS11	LC599405.1

**Table 4:** Biomass and biofilm formation assays for 4 bacterial consortia exposed to cyantraniliprole at concentrations of 60, 70, 80, and 90 mg/L (OD measured at _600_ nm)

Pesticides Conc.	Biomass Was Determined at OD_600_ nm, and Biofilm Formation at OD_570_ nm.											
	70 mg/L		80 mg/L		90 mg/L		100 mg/L		ODNC *		ODPC **	
Bacterial Strains	Biomass	Biofilm	Biomass	Biofilm	Biomass	Biofilm	Biomass	Biofilm	Biomass	Biofilm	Biomass	Biofilm
Consortia No. 1	2.322 ^d^	0.689 ^d^	2.231 ^d^	0.638 ^d^	1.789 ^c^	0.611 ^d^	1.587 ^d^	0.521 ^d^	0.059	0.067	1.289 ^d^	0.557 ^d^
Consortia No. 2	2.344 ^c^	0.722 ^c^	1.242 ^c^	0.656 ^c^	1.811 ^d^	0.622 ^c^	1.632 ^c^	0.531 ^c^	0.058	0.067	1.352 ^c^	0.589 ^c^
Consortia No. 3	2.323 ^b^	0.742 ^b^	2.255 ^b^	0.663 ^b^	1.831 ^b^	0.647 ^b^	1.641 ^b^	0.552 ^b^	0.059	0.067	1.368 ^b^	0.611 ^b^
Fahmy-Consortia	2.388 ^a^	0.792 ^a^	2.317 ^a^	0.687 ^a^	1.876 ^a^	0.682 ^a^	1.662 ^a^	0.593 ^a^	0.057	0.067	1.474 ^a^	0.642 ^a^

Different letters represent significant differences (Duncan’s test, *p* < 0.05) among all treatments. * The mean OD value of negative controls (ODNC) was 0.057 to 0.059 ± 0.005 for the biomass assay and 0.067 ± 0.005 for biofilm formation. ** The mean OD value of positive controls (ODPC) was 1.289 to 1.474 ± 0.119 for the biomass assay and 0.557 to 0.642 ± 0.072 for biofilm formation.

**Table 5:** Decolorization time (hours) of 0.1% DCPIP indicator in Bushnell-Haas broth by bacterial consortia over a 72 h experiment

Bacterial Strain	Time for Decolorization of DCPIP During the Experiment (72 h).	Relative Biodegradation Activity *
	Cyantraniliprole	
Control	No Decolorization	No Activity
Consortia (No. 1)	27 ^a^	Moderate
Consortia (No. 2)	23 ^b^	High
Consortia (No. 3)	20 ^c^	Very high
Fahmy-Consortia	17 ^d^	Highest

* Shorter DCPIP decolorization time indicates greater metabolic activity and stronger Cyantraniliprole biodegradation potential. Different letters represent significant differences (Duncan’s test, significant difference test at *p* < 0.05) among all treatments.

**Table 6:** Growth and metabolic activity of four selected bacterial consortia (Nos. 1, 2, and 3) in Mineral Salt Medium supplemented with different diamide insecticides (100 mg/L) at 35 °C.

Bacterial Strain	Various Diamide Insecticides (Bacterial Growth at 600 nm)				
	Cyantraniliprole	Flubendiamide	Chlorantraniliprole	Cyclaniliprole	Fluchlordiniliprole
Consortia (No. 1) ^1^	2.144 ^d^	2.115 ^d^	2.166 ^d^	2.198 ^d^	2.112 ^d^
Consortia (No. 2) ^2^	2.176 ^c^	2.181 ^c^	2.171 ^c^	2.241 ^c^	2.135 ^c^
Consortia (No. 3) ^3^	2.188 ^b^	2.217 ^b^	2.262 ^b^	2.252 ^b^	2.145 ^b^
Fahmy-Consortia ^4^	2.211 ^a^	2.230 ^a^	2.272 ^a^	2.262 ^a^	2.216 ^a^

Different letters represent significant differences (Duncan’s test, significant difference test at *p* < 0.05) among all treatments.

**Table 7:** Biodegradation of Cyantraniliprole added at 60 mg/L in water and recovery (zero-day) tests with bacterial consortia during the incubation periods at 30 °C for 20 days, as determined by HPLC in water.

Treatments	60 mg/L Degradation/500 mL Water					
	Recovery	Zero-Day	3-Days	7-Days	11-Days	20-Days
C1	96.60	57.96	55.33 ^a^	51.76 ^a^	47.36 ^a^	40.99 ^a^
C2	96.54	57.92	54.41 ^b^	50.34 ^b^	46.88 ^b^	39.90 ^b^
T1	96.65	57.99	45.88 ^c^	34.89 ^c^	25.60 ^c^	4.64 ^c^
T2	96.47	57.88	44.22 ^d^	33.11 ^d^	22.31 ^d^	3.30 ^d^
T3	96.48	57.89	43.13 ^e^	31.14 ^e^	19.52 ^e^	1.90 ^e^
T4	96.32	57.79	42.56 ^f^	29.07 ^f^	17.21 ^f^	1.00 ^f^

**Recovery% (96.32–96.60).** The control groups included **C1** (cyantraniliprole-contaminated water without NPK or inoculum) and **C2** (contaminated water with NPK but no inoculum). Treatments **T1–T4** exposed cyantraniliprole-polluted water to NPK and distinct bacterial consortia: **T1** (Consortium No. 1), **T2** (Consortium No. 2), **T3** (Consortium No. 3), and **T4** (Consortium No. 4, Fahmy consortium). Significant differences among treatments (denoted by letters) were determined using Duncan’s test (*p* < 0.05).

**Table 8:** Bioinformatic analysis-based bacterial contribution is potentially activated in the different bacterial consortia during the biodegradation of Cyantraniliprole.

Enzymes	TPI	AP	C1,2O	COMT	OEX
Consortia No. 1					
*B. subtilis* subsp. *subtilis* Re1	✓	✓	✓	✓	X
*B. cereus* Re2	✓	✓	✓	X	✓
*Bacillus pumilus* Re3	✓	✓	✓	✓	X
*Bacillus paramycoides* Re4	✓	✓	X	X	X
*Bacillus mycoides* Re5	✓	✓	X	X	X
*B. amyloliquefaciens* Re6	✓	✓	✓	X	X
Total enzymes of consortia No. 1 ✓ = 19 enzymes					
Consortia No. 2					
*Serratia marcescens* BO1	✓	✓	✓	X	X
*Serratia marcescens* BO2	✓	✓	✓	X	X
*Pseudomonas aeruginosa* BO3	✓	✓	✓	X	✓
*Pseudomonas aeruginosa* BO4	✓	✓	✓	X	✓
*Pseudomonas aeruginosa* BO5	✓	✓	✓	X	✓
*pseudomonas aeruginosa* BO6	✓	✓	✓	X	✓
Total enzymes of consortia No. 1 ✓ = 22 enzymes					
Consortia No. 3					
*Bacillus subtilis* Ht1	✓	✓	✓	✓	X
*Bacillus subtilis* Ht2	✓	✓	✓	✓	X
*Bacillus mojavensis* Ht3	✓	✓	✓	✓	X
*pseudomonas aeruginosa* Ht4	✓	✓	✓	X	✓
*Pseudomonas aeruginosa* Ht5	✓	✓	✓	X	✓
*Pseudomonas aeruginosa* Ht6	✓	✓	✓	X	✓
Total enzymes of consortia No. 1 ✓ = 24 enzymes					
Consortia No. 4 (Fahmy consortia)					
*Bacillus subtilis* subsp. *subtilis* AZFS3	✓	✓	✓	✓	X
*Bacillus pumilus* AZFS5	✓	✓	✓	✓	X
*Bacillus mojavensis* AZFS15	✓	✓	✓	✓	X
*Bacillus paramycoides* AZFS18	✓	✓	X	X	X
*Pseudomonas aeruginosa* KZFS4	✓	✓	✓	X	✓
*Alcaligenes aquatilis* KZFS11	✓	✓	✓	✓	X
Total enzymes of consortium No. 1: ✓ = 22 enzymes					

TPI, Triosephosphate isomerase. AP, Aminopeptidase. C1,2O, Catechol 1,2-dioxygenase. COMT, Catechol O-methyltransferase. OEX, 2-oxopent-4-enoate hydratase. The entry ✓ refers to gene activation while X refers to inactivity. Consortia No. 1 contributed 19 enzymes, covering all 5. Consortia No. 2 contributed 22 enzymes, covering all 4 enzymes except Catechol O-methyltransferase (COMT). Consortia No. 3 contributed 24 enzymes, covering all 5. Consortia (T4) exhibited the most robust enzymatic activity, with 22 enzymes.

## Data Availability

Data will be available upon request.
